# Social inclusion and exclusion of people with mental illness in Timor-Leste: a qualitative investigation with multiple stakeholders

**DOI:** 10.1186/s12889-019-7042-4

**Published:** 2019-06-07

**Authors:** Teresa Hall, Ritsuko Kakuma, Lisa Palmer, Harry Minas, João Martins, Michelle Kermode

**Affiliations:** 1 Nossal Institute for Global Health, University of Melbourne, 333 Exhibition St, Melbourne, Victoria 3004 Australia; 2 Centre for Global Mental Health, London School of Hygiene and Tropical Medicine, London, UK; 3 Centre for Mental Health, University of Melbourne, Melbourne, Australia; 4 School of Geography, University of Melbourne, Melbourne, Australia; 5 Faculty of Medicine and Health Sciences, National University of Timor-Leste, Díli, Timor-Leste

**Keywords:** Social inclusion, Human rights, Global mental health, Low-and middle-income country, Health policy and systems research, Timor-Leste

## Abstract

**Background:**

Social inclusion is a human right for all people, including people with mental illness. It is also an important part of recovery from mental illness. In Timor-Leste, no research has investigated the social experiences of people with mental illness and their families. To fill this knowledge gap and inform ongoing mental health system strengthening, we investigated the experiences of social inclusion and exclusion of people with mental illness and their families in Timor-Leste.

**Methods:**

Eighty-five participants from the following stakeholder groups across multiple locations in Timor-Leste were interviewed: (1) people with mental illness and their families; (2) mental health and social service providers; (3) government decision makers; (4) civil society members; and (5) other community members. Framework analysis was used to analyse interview transcripts.

**Results:**

People with mental illness in Timor-Leste were found to face widespread, multi-faceted sociocultural, economic and political exclusion. People with mental illness were stigmatised as a consequence of beliefs that they were dangerous and lacked capacity, and experienced instances of bullying, physical and sexual violence, and confinement. Several barriers to formal employment, educational, social protection and legal systems were identified. Experiences of social inclusion for people with mental illness were also described at family and community levels. People with mental illness were included through family and community structures that promoted unity and acceptance. They also had opportunities to participate in activities surrounding family life and livelihoods that contributed to intergenerational well-being. Some, but not all, Timorese people with mental illness benefited from disability-inclusive programming and policies, including the disability pension, training programs and peer support.

**Conclusions:**

These findings highlight the need to combat social exclusion of people with mental illness and their families by harnessing local Timorese sociocultural strengths. Such an approach could centre around people with mental illness and their families to: increase population mental health awareness; bolster rights-based and culturally-grounded mental health services; and promote inclusive and accessible services and systems across sectors.

**Electronic supplementary material:**

The online version of this article (10.1186/s12889-019-7042-4) contains supplementary material, which is available to authorized users.

## Background

Social exclusion of people with mental illness remains an unresolved global public health and human rights challenge [1]. Social exclusion and social inclusion are not simply the inverse of each other, and there is no consensus on definitions [2]. We adopt the World Health Organisation (WHO) definition of social exclusion as: “*the dynamic, multi-dimensional processes driven by unequal power relationships interacting across four main dimensions - economic, political, social and cultural - and at different levels including individual, household, group, community, country and global levels.*” [3]. We define social inclusion in relation to the right to full participation in society espoused through the United Nations Convention on the Rights for Persons with Disabilities (UNCRPD) [4]. That is, social inclusion involves feeling accepted, having individual and collective agency to determine participation, and the removal of structural and attitudinal barriers to participation [5, 6].

Stigma is a key determinant of social exclusion [3]. Stigma is a social and subjective process involving problems of knowledge, prejudicial attitudes, and discriminatory behaviour [7–9]. Across countries, stigmatising beliefs often cast people with mental illness as dangerous, unpredictable and unintelligent; beliefs which are enacted through discriminatory and exclusionary behaviours [10–12]. Globally, many people with mental illness are excluded from employment (economic exclusion), denied legal rights to vote, marry or own land (political exclusion), and ostracized (sociocultural exclusion) [13–16].

Stigma, discrimination and social exclusion have deleterious effects on people with mental illness. Stigmatising attitudes towards people with mental illness are linked to poor well-being and self-esteem [17]. Mental illness and social isolation are both linked with early death through the direct and indirect pathways of chronic disease and lifestyle factors [18, 19]. Mental illness stigma and discrimination also impede help-seeking and access to health care, which inhibits recovery [11].

Promoting social inclusion of people with mental illness is consequently a key goal of human rights and global mental health programming to achieve people-centred mental health care [20]. Interventions to promote social inclusion aim to minimize the impact of attitudinal, structural and behavioural drivers of social exclusion. In high-income countries (HICs), social inclusion of people with mental illness has been promoted through community-based mental health, employment and housing services, legislative protections, and anti-stigma interventions [21]. There is good evidence that supported employment programs for people with mental illness and interventions to reduce mental health stigma (e.g. mental health education, direct contact with people with mental illness) are effective in HICs [22, 23].

However, research about social inclusion of people with mental illness (i.e. theory, prevalence, experiences of, and interventions) from low- and middle-income countries (LMICs) is sparse, which is problematic because explanatory models and consequences of mental illness, and the extent of stigma and social exclusion, differ across cultures [6, 24]. The communitarian orientation of many LMICs locates responsibility for the individual, their illness and stigma with the family [24–26]. There is an emerging body of evidence related to social inclusion and exclusion of people with mental illness in Asia, but only a few studies have examined the perspectives of people with mental illness and their families, and even fewer have explored positive effects of inclusion [27–30].

In Timor-Leste, a LMIC in South-East Asia, the limited available research about mental health has focused primarily on health service access, illness prevalence, and customary healing [25, 31, 32]. The recently approved National Mental Health Strategy Timor-Leste 2018–2022 strives for a comprehensive people-centred mental health system, including programming to eliminate stigma and discrimination towards people with mental illness [33]. This study aims to investigate the experiences of and opinions about social inclusion and exclusion of Timorese people with mental illness from the perspective of multiple stakeholders. This is essential information for developing programs that promote social inclusion relevant to the local Timorese sociocultural context, and also for providing a starting point for deeper theoretical consideration on the topic grounded in LMIC knowledge.

## Methods

Individual and group interviews with a range of stakeholders were conducted in communities, health facilities and workplaces. Data were collected between September 2017 and August 2018 as part of TH’s PhD project investigating people-centred mental health care in Timor-Leste. This research was intended to inform the implementation of the National Mental Health Strategy Timor-Leste 2018–2022 [33]. Ethical approval was obtained from University of Melbourne Human Ethics Sub-Committee (1749926) and National Institute of Health in Timor-Leste (1070MS-INS/DE-DP/CDC-DEP/IX/2017).

### Study setting

Timor-Leste is a culturally and ethnically diverse nation of 1.3 million people [34]. It is one of the poorest countries in South East Asia, with 70% of Timorese living in rural areas and reliant on subsistence farming [35, 36]. According to the 2015 census, approximately 1.3% of the Timorese population have a mental illness [37], but this is likely to be an underestimation given that 2016 Global Burden of Disease estimates a 11.6% prevalence of mental and substance use disorders [38]. Securing human rights is a key focus of the Timorese government and civil society, in line with the international rights-based agenda prioritised since Timor-Leste’s independence from Indonesia in 1999 [39]. More specifically, emphasis has recently been placed on the rights of people with disabilities as Timor-Leste moves towards ratifying UNCRPD. A government pension is available to families who have a member(s) with disability, although 86% of people with disability in Timor-Leste do not receive this pension [40].

This study investigated social inclusion and exclusion of people with mental illness in community, health and social settings across national and sub-national administrative levels in Timor-Leste. The study sites were Dili, Baucau, Venilale and Laclubar. Dili was selected because it is the capital and a hub for government, civil society organisations, and mental health service providers including the largest mental health non-government organisation (NGO), Pradet. The availability of mental health care in Timor-Leste is limited, and mostly delivered through primary health centres and posts, and hospitals in each municipality [32, 33]. Baucau municipality and its administrative post Venilale were selected as locations to explore the experiences of rural-dwelling people with mental illness. Baucau and Venilale populations provide a fair representation of Timorese life. Each have: on average 5.5 people per household; employment rates around 50%; a 72% enrolment rate in secondary school in Baucau around the national average of 76%; and youth literacy around the national average of 84% [37]. Baucau is a coastal municipality in Eastern Timor-Leste with a population of 123,203 people, and a municipality referral hospital that provides mental health care [37]. Venilale is a mountainous rural township with a mental health nurse working in the local government health clinic. We also included Laclubar administrative post in Manatuto municipality because it hosts the country’s only inpatient mental health facility run by NGO, São João de Deus.

### Participants

Eighty-five participants (≥18 years) were interviewed representing the following stakeholder groups: (1) people with mental illness and their families (*n* = 30, 2) mental health and social service providers (*n* = 23, 3) government decision makers (*n* = 10, 4) civil society members (*n* = 9; and (5) other community members and organisations (*n* = 13) (see Table 1). This article foregrounds the voices and experiences of people with mental illness and their families. All participants with mental illness were consulting (or had so previously) government health services in Venilale and Baucau, and NGO services in Dili for their mental health problems. People with mental illness and their families were recruited through these service providers known to them, introduced to TH, and invited to participate in the study. TH recruited other stakeholders by contacting them directly via phone, email or in-person, introducing the purpose and requirements of the study, and inviting them to participate. Interviews with other stakeholders provided a range of perspectives about the extent of inclusion and exclusion.

**Table 1:** Participant demographics

	People with mental illness		Family members		Service providers		Decision makers		Civil society		Other community members and organisations		Total	
N	20		10		23		10		9		13		85	
	n	%	n	%	n	%	n	%	n	%	n	%	N	%
Age														
26–40	12	60	2	20	10	43.5	1	10	4	44.4	6	46.2	35	41.2
41–55	6	30	5	50	8	34.8	8	80	3	33.3	5	38.5	35	41.2
56–70	2	10	3	30	5	21.7	1	10	2	22.2	2	15.4	15	17.6
Gender														
Male	7	35	7	70	13	56.5	9	90	8	88.9	7	53.8	51	60.0
Female	13	65	3	30	10	43.5	1	10	1	11.1	6	46.2	34	40.0
Education														
None	1	5	2	20	0	0.0	0	0	0	0.0	0	0.0	3	3.5
Primary	11	55	5	50	0	0.0	0	0	0	0.0	0	0.0	16	18.8
Secondary	4	20	1	10	1	4.3	0	0	4	44.4	3	23.1	13	15.3
Tertiary	4	20	2	20	22	95.7	10	100	5	55.6	10	76.9	53	62.4
Location														
Dili	5	25	0	0	15	65.2	5	50	6	66.7	9	69.2	40	47.1
Baucau	2	10	1	10	4	17.4	4	40	0	0.0	3	23.1	14	16.5
Venilale	13	65	9	90	3	13.0	1	10	3	33.3	1	7.7	30	35.3
Laclubar	0	0	0	0	1	4.3	0	0	0	0.0	0	0.0	1	1.2

Notes: We adopt WHO’s definition of civil society as individuals and organisations working for “*collective action around shared interests, purposes and values, generally distinct from government and commercial for-profit actors*” [62]. Civil society includes community groups, social movements and advocacy groups. Civil society also includes local chiefs and customary healers who may not be mobilised in formal groups. Other community members and organisations include representatives from international development agencies, law enforcement, universities, and other people with relevant knowledge but who do not work specifically in mental health in Timor-Leste

### Data collection

For the PhD project, interview guides for each type of participant were semi-structured and aligned with the WHO Framework on Integrated People-Centred Health Services (WHO IPCHS) [41]. This article focuses on questions about the daily experiences, activities, and health and social care access for people with mental illness and their families. The interview guides were translated, their meaning checked by an independent interpreter, and piloted with three Timorese colleagues before data collection commenced. TH conducted interviews directly in English (*n* = 25), or with a trained interpreter in the national languages Tetum (*n* = 48) or Portuguese (*n* = 1). Several interviews were conducted in two of the local languages of Venilale and Baucau and then simultaneously translated into English (Makassai: *n* = 7, Cairui: *n* = 4). Interviews were audio recorded with participant consent. Interviews lasted on average 47 min (*range*: 7 to 111 min) and were conducted in communities, health facilities and workplaces. Consistent with local cultural norms and upon request, most participants with mental illness had a family member or other known person present during their interviews to foster a comfortable environment (*n* = 16, 70% male family member) [25]. Audio-recorded interviews were transcribed in English (verbatim or translated sections) and checked for meaning by an independent translator. See Additional file 1 for full interview guides in English and Tetum languages.

### Data analysis

TH analysed interview data using NVivo 9 software [42]. Framework analysis is an inductive and deductive analysis method [43] that we used to extract data relevant to the overall a priori research topic (people-centred mental health care) including the current article (social inclusion), while allowing new themes to emerge [43]. Framework analysis consists of five stages: (1) transcription; (2) familiarisation with the data; (3) coding of a priori and emergent themes; (4) preliminary analytical framework; and (5) applying the analytical framework. TH developed and applied a preliminary coding framework to five transcripts. A priori themes were structured around the five key domains of people-centred health care in the WHO IPCHS (empowerment, participation, health service model, intersectoral collaboration and enabling environment) [41]. After discussion and refinement with MK, the final analytical framework identified three main themes and 17 sub themes related to inclusion and exclusion of people with mental illness in Timor-Leste.

## Results

Findings are structured using the sociocultural, economic and political dimensions of the WHO definition of social exclusion [3]. Findings on access to health are reported elsewhere [44]. Participant quotes are labelled as: person with mental illness (PWMI); family member (FM); service provider (SP); decision maker (DM); civil society member (CS); and other community member (OT). Table 2 presents the themes and sub themes of social inclusion and exclusion.

**Table 2:** Dimensions of social inclusion/ exclusion, sub themes and example quotations

Dimension	Sub theme	Participant	Example quotation
1. Socio cultural	1.1 Explanatory model of mental distress	Family member (64 years, male)	Most of [my wife’s] problems are from the Indonesian occupation
		Service provider (41 years, female)	[The woman’s parents] didn’t want to give the medicine to [her] because if she got better another family member might get sick with the same problem.
		Decision maker (51 years, male)	Some people who are crazy become normal again when they go back [and tend to] their sacred houses.
	1.2 ‘crazy’, Dangerous	Person with mental illness (53 years, female)	Before I took medications, I would just walk around, and just yell, just scream, just swear. And if someone walked by and stared at me, I would just swear at them. And that’s how people around here knew that something was wrong with me.
		Family member (42 years, male)	He was very aggressive, he tried to [throw] stones at people, and destroy everything.
		Service provider (35 years, female)	The mother [with mental illness] was hitting her stomach when she was pregnant and when the child was born. After one month, the mother was beating [the child].
	1.2 Capacity and abilities	Person with mental illness (29 years, female)	Even when I went to Dili [when I was unwell], my brain was still normal. I could still decide what my objectives were and where to go and find a solution. It is just that I felt afraid, felt scared.
		Civil society (29 years, female)	People with mental illness have a problem with their mind so everything they do is not right
		Decision maker (46 years, male)	People with mental health problems cannot discuss or think properly
	1.2 Incurability and shame	Service provider (58 years, male)	Also the majority of the community seems to believe that you can’t heal or treat mental health problems
		Civil society (62 years, male)	It could be that the family feel shame, yes. Another thing is that the family believe the illness is from their ancestors, generations, so they think that once [the person] is crazy, it is just going to be like that.
		Civil society (30 years, male)	[Having a good image in the community is] such a core of someone’s values and beliefs that it could actually destroy you, the shame [from having mental illness in your family] could actually destroy you
	1.3 Altered social roles	Service provider (38 years, female)	Every time she went to her sister’s house they wouldn’t look after her. So she just walked around the streets.
		Decision maker (48 years, male)	For example, if there is a crazy person and he is aggressive and destroys things, it means that the family have to look after him every day. He can’t go to work, he is crazy. It will stop the family from doing things, like work.
	1.3 Discrimination	Family member (64 years, male)	With the neighbours and the community, sometimes when they come and talk to her, they say something wrong [“crazy”], and she will get angry. But with my children, I always let them know not to do anything that will make her angry.
	1.3 Vulnerability	Civil society (42 years, male)	People with mental disabilities [are] very easy for other people to influence, and do other things to them
	1.3 Violence	Family member (44 years, male)	[When my wife was sick], she was always arguing and abusing me. […] Once we had a very big argument and she was hitting me and so I hit her and the police came because she was bleeding.
		Civil society (42 years, male)	[A man from Liquisa municipality] was killed in Attaby because he destroyed the water pipe, he broke it. So the community – you know, water is very important – so the community were aggressive and they killed him. He was killed on the street.
	1.3 Confinement and chaining	Family member (46 years, male)	Man: yes, I chained [my brother] three times. The first was when I was working with the UN so they gave me handcuffs and I put them on him. But after that, he used a saw to cut them. At that time, I was away but when I came back I tied him up again with rope but then he cut it and untied himself. I went to the police and asked for another set of handcuffs to use on him, and then somehow seven or eight months later he cut those too. I went once again to the police and they said, “no more”. TH: would you prefer to chain him or that he is unchained? Man: only when he is sick. At the time he always beat my children, and once he was trying to throw a stone at my Mum. And he was trying to kill me, so I decided to chain him up
		Civil society (29 years, female)	[People] think that it is better to keep [people with mental illness in the house […] and leave them in the house for the rest of their lives. Because [people] think that if they let [people with mental illness] go, they might cause some problems outside.
		Decision maker (46 years, male)	The main cause is shame, when one member of the family has a mental illness, [the family] feel shame, that is why they isolate the [unwell person]. Sometimes they are tied up or far away from home or isolated.
	1.4 Acceptance	Person with mental illness (53 years, female)	It is family that is more important to me. Because with family, even if I am sick, they are the ones that will look after me. Because we have problems to face, economical problems, but if I have my family everything is ok. So family is like number one. Because even when I am sick, my family will look after me.
		Family member (42 years, male)	People who live in this area are our family … At first when [my sister] got sick, [the community] were afraid of her, but they still looked after her and saw her as a daughter and a relative. When she returned from [the mental health facility in] Laclubar, the doctors said that she has to stay in a quiet place with no distractions. So when people are making noise near our house, our neighbours are the ones who help us to tell those people to stay away or be quiet.
	1.4 Recovery	Person with mental illness (36 years, male)	Because when I dance [at Pradet] it reminds me of when I was little, before I used to be involved in dancing and I used to use traditional tais for the dancing.
		Decision maker (46 years, male)	Participation [in the community] is just for people who have recovered can participate but those ones that haven’t, cannot.
	1.4 Peer support	Person with mental illness (31 years, female)	I don’t talk [about my difficulties] when other people ask me or talk to me about it […] because I am afraid they might break my heart or make me feel bad so I don’t talk to them about it. I am open with my friends [at Pradet], we are open with each other.
2. Economic	Contribution	Family member (60 years, female)	[my daughter has] only [studied until] 6th grade. If she can get better again, then it would be good for her to stay here and help our family with [domestic] work
		Civil society (42 years, male)	[Mental illness] destroys the whole family. I’m trying to say that if the head of the family is mentally ill, they have lost the key resources of the family. They lose everything. Particularly for families who are very poor. Unemployment happens, there is nothing there, they only rely on the farm to grow corn of cassava or vegetable so [they] need that person.
	Employment	Person with mental illness (53 years, female)	I really like to take medicines [… because] I am doing my sewing again, so I can sell items and get some money [to buy my childrens’ school uniforms] and I also sell stuff to support my community.
		Person with mental illness (31 years, female)	I would love to work at an organisation or at the government or in an office[…] I would like to work as a normal person.
		Service provider (41 years, male)	[employers] are going to get tired when in the third week the person [with mental illness...] stops turning up to work, or starts shouting at one of the other staff or one of the customers.
3. Political	3.1 Education	Person with mental illness (29 years, male)	After the injections, I seemed to lose my mind. I couldn’t remember things.
		Family member (42 years, male)	We [the family] have been thinking about any training that she could get, like training for sewing or to do something but we don’t who we need to contact [to organize this]? We would like her to develop herself. She needs an activity that could help her to think. She couldn’t just sit like this [at home all day].
	3.2 Social protection	Family member (46 years, male)	The government gives the subsidy to one of my brother’s [with mental illness], but not my other [brother …] with my brother who gets the subsidy, at the time he was ok with organising all his documents. He was willing to go to the health centre for them to assess whether he has a mental health problem or not. But with my other brother, when we were trying to organise his documents, the health staff required that we bring him to the health clinic so they could assess him, but then my brother didn’t want to go so we couldn’t organise it.
	3.3 Legal and political representation	Other community member (46 years, male)	If [the crime] is committed against their own family then it is ok, but if they commit a crime towards other people then sometimes that person wants to put the person with the mental problem in jail. But it is a very hard decision. The law is to put [anyone who commits a crime into jail.
		Civil society (29 years, female)	We haven’t had any people who have recovered from their mental illness who want to be involved in the [disability support] group.

### Sociocultural exclusion and inclusion

Sociocultural inclusion refers to connections with family and community and acceptance of diverse ways of being and living.

### Explanatory models of mental distress

Explanatory models of mental distress influenced the way that people with mental illness and their families were received in Timorese society. Participants attributed mental distress to multiple causes. The explanatory belief most frequently reported, including by people with mental illness, was that mental distress resulted from a spiritual imbalance, originating from ancestors, that the current generation had to address through cultural practices. One woman said her brother was unwell because, “*there is something wrong in our father’s family*” (FM, 26–30 years, female). Locating responsibility for the cause, treatment and any distress-behaviours with the family, attached shame to them: *“Sometimes the family is not open [about having mental illness in the family] to the community or the local leaders [… because they believe the illness] is punishment from the ancestors*” (OT, 36–40 years, male). The second most commonly reported explanatory belief was that mental distress was caused by trauma related to the Indonesian occupation: “*A lot of bombs exploded, that’s what made her afraid and traumatised*” (FM, 30–35 years, female). Participants also attributed mental illness to stress stemming from economic instability, problematic or violent relationships, or visiting natural sites (e.g. land, water sources) without spiritual permission.

### Beliefs about people with mental illness

Most participants across all stakeholder groups understood the term ‘mental illness’ in reference to people with severe mental illness, and used the Tetum word for crazy, *“bulak”.* Participants defined *bulak* as someone who behaved in strange, aggressive or dangerous ways e.g. walking and talking alone, destroying property or shouting at strangers. People with mental illness were commonly deemed by participants to lack cognitive capacity and so, *“ need full assistance from their family and everyone because [they cannot] take care of themselves”* (CS, 26–30 years, female). Some people with mental illness were also seen to be incurable and hence considered “*crazy until death”* (CS, 55–70 years, male). This was often the conclusion when illness was attributed to ancestral causes, or when the unwell person had not recovered after receiving traditional or medical treatment.

### Discrimination and violence

Discrimination and violence against people with mental illness in Timor-Leste was widely described. People with mental illness and other participants described how community members, particularly children, had thrown stones or heckled them yelling “*crazy crazy*”. Some participants with mental illness reported that their community and family thought they were lazy, useless, of bad character and had brought shame on the family. One young man with mental illness stated: “*I don’t know what I did wrong, but I think [the community] think I’m not good, that’s why they beat me. But I’ve never done anything wrong*.” (PWMI, 26–30 years, male). Some women with mental illness were deemed dangerous by their families’ and therefore removed from child rearing responsibilities. One civil society member cautioned that the label of mental illness sometimes endured despite recovery:

Some people have recovered from their illness but the community is still bullying them, which makes them become aggressive again and then they relapse. (CS, 40-44 years, male)*.*

Extreme violence was also reported against people with mental illness. This included the fatal shooting of a man with mental illness by a policeman, and sexual violence against women with mental illness. Several civil society and government members stated that perpetrators justified their non-consensual sex with women with mental illness on the grounds that the women would not know or understand, or they were like “*pig [s]*” (CS, 40–44 years, male).

Many participants believed that violent experiences could “*provoke*” people with mental illness to become violent, unwell or relapse. One elderly man with mental illness who lived alone explained: “*Normally kids come and try to annoy me and say bad words. And then I get angry and go after them so that they get scared and leave.*” (PWMI, 70–74 years, male). Participants also reported (and TH saw) that wooden planks and machetes were sometimes used to punish people with mental illness for transgressions against community members.

People with mental illness were often confined to the home because they were believed to be at risk from other community members, or conversely, they themselves posed a threat. Some families said they employed chains and shackles to ensure that their unwell relative did not run away. Restraining the person was seen as preferable to abandoning them because they at least remained within the family.

### Facilitators of social inclusion: acceptance, recovery and peer support

While reports of social exclusion were common, experiences of social inclusion were also described. All participants with mental illness lived with or near their families. They reported that family and community connectedness helped them to feel protected and accepted. One woman with mental illness reported being entrusted with social tasks: “*I feel included because every time the community have some activities, they will invite me to cook*” (PWMI, 36–40 years, female). One rural-dwelling man with mental illness said: *“[the community] always look after me, they never say anything [bad]*.” (PWMI, 20–25 years, male).

Friendships were important for many of the participants with mental illness. Several service users of the national mental health NGO, Pradet, described the support and acceptance that they felt from peer service users: “*we are all friends we give each other courage…we really respect each other… we are a very strong team, we do it together.”* (PWMI, 30–35 years, female). Several participants in Dili identified the need for peer support groups for people with mental illness to feel supported and conduct mental health awareness raising.

Spirituality and religious practice were another vehicle for social inclusion of people with mental illness. One woman with mental illness described how she found peace by attending Catholic mass and praying. When asked why she felt attending mass was important, she said: *“I don’t think of anything else when I am praying and reading the bible. It helps my brain and everything to be quiet with God […] So it helps me*.” (PWMI, 30–35 years, female).

### Economic exclusion and inclusion

Economic inclusion refers to employment and livelihoods. Some participants reported that people with mental illness encountered barriers when trying to access and remain in formal employment. The barriers were sometimes a product of the illness. Recounting how his wife had stopped teaching for 3 years while she was unwell, a man explained that the school “*supported her but she abused them and was always yelling and shouting at people*.” (FM, 40–45 years, male). Other participants were fearful of being stigmatised so hid their illness from their employer because they were afraid that they would lose their job or be treated as “*a joke*” (OT, 30–35 years, female).

Lack of economic participation compromised individual and family well-being. Several participants explained that families experienced financial hardship when their primary income producer could not work because of ill-health. One young man who had recovered described his worries:

I worry a lot because some of my friends are police, some of them are in the army, and some of them have already visited other countries. And I'm still here [in our hometown] and I want to have a good future but I don't know [how]. (PWMI, 26-30 years, male)*.*

Examples of economic inclusion were also described. Many participants with mental illness described undertaking domestic or farming tasks i.e. looking after animals, sweeping the house or selling vegetables, which are common livelihood activities in rural Timor-Leste. They felt that contributing to their families in this way symbolised recovery and helped them to remain well: “*when I feel better again I want to work in the rice field*” (PWMI, 20–25 years, male). Some participants with mental illness had recovered fully and returned to work. One teacher in a rural area said “*moral support”* from the school leaders and fellow teachers allowed her to return to work after time off when she was unwell (PWMI, 26–30 years, female). In Dili, a Pradet service user established his own vegetable plantation, which was subsequently incorporated into Pradet’s programming.

Contributing to family livelihoods mitigated the stigma of mental illness and facilitated inclusion (see Table 2). One man described his perception that the community would reciprocate positive contributions he made*: “I feel better now. With the community, if I help them they will be good with me and if not, then they won’t be.”* (PWMI, 26–30 years, male). A civil society member described how one woman was a “*good crazy person*” because she “*can still do things, like cook for her husband.*” (CS, 60–65 years, male).

### Political exclusion and inclusion

Political inclusion refers to access to public services including education, social protection and legal representation.

### Education

Participants reported that mental illness contributed to reduced educational attainment. Approximately half of the participants with mental illness had attended secondary school, with the duration of their attendance curtailed by the onset of their illness and lack of family resources. Many participants, particularly in rural areas, discussed their and their family member’s withdrawal from school or tertiary study due to mental illness. One woman described how her brother’s withdrawal from school had precipitated his illness: “*So at the time, he was studying grade 6 but when he dropped out of school, he started to lose his mind*.” (26–30 years, female). Illness experiences and medication side effects were reported to reduce concentration: “*my hands and feet were shaking and then I lost my memory so I couldn’t think of anything*” (PWMI, 30–35 years, female). One educator described how a female student with mental illness could not continue to study because she was “*a little bit dangerous*” and could not “*cooperate and […] participate in class*” (OT, 35–40, male). These reduced educational opportunities prevented many people with mental illness from gaining the necessary knowledge and skills for employment or passing milestones important to them: “*I really want to [study] because I see that all my other friends are getting their Bachelor degrees and it makes me a bit stressed*.” (PWMI, 30–35 years, female).

Some participants explained that the development of the one-year Community Based Rehabilitation (CBR) diploma at the National University of Timor-Leste [45] provided a pathway for some people with mental illness to study alongside people with other disabilities and social service providers. At the time of data collection, three people with self-declared mental illness had enrolled in this course, and one person had completed it. Currently, efforts by the Ministry of Education to implement disability-inclusive primary and secondary education do not include young people with mental illness, but government representatives identified this as a future priority.

### Social protection

The government pension for families affected by disability is available for people with mental illness. To access this entitlement, families require valid identity cards, and a health and social assessment by the Ministries of Health and Social Solidarity and Inclusion respectively. This is a lengthy process that requires families to navigate formal systems only available in the townships. Most families interviewed did not receive the social protection subsidy. One health educator said that many families did not know about the disability subsidy. Given the competing economic demands on poor families, one community member questioned whether *“the family use this money to attend [to their unwell family member] or not.*” (OT, 36–40 years, male). Some families said they had received donations of rice from various government and NGO sources.

### Legal and political representation

Civil society members explained that their advocacy for disability rights included people with mental illness, but they elaborated that people with mental illness were not actively involved in the disability sector because there were no public advocates with mental illness. These participants also indicated that people with mental illness were unlike people with other disabilities, who had *“normal*” cognitive capacity (CS, 26–30 years, female). One human rights activist said that there was confusion amongst decision makers and election adjudicators as to whether people with mental illness had the right to vote.

Participants reported that people with mental illness were sometimes in contact with the criminal justice system as victims or perpetrators of crime. Because of their perceived lack of capacity, participants explained that people with mental illness were unable to provide valid testimony:

Normally if the crazy people inform local authorities or anyone [in the community about their problems], no one will notice (laughing)… because most people know that they are crazy. (CS, 60-65 years, male)*.*

In both Dili and rural areas, legal support providers said they were unable to represent clients with mental illness because they could not communicate with them. Participants reported inconsistent practices for the prosecution of crimes involving defendants with mental illness. Several participants said that people with mental illness were sent to jail, whereas other participants maintained that people with mental illness were not penalized for crimes because of their illness.

## Discussion

We report here an investigation of multiple perspectives on the social inclusion and exclusion of people with mental illness in Timor-Leste. People with mental illness in Dili, Baucau, Venilale and Laclubar faced widespread sociocultural, economic and political exclusion. Nonetheless, experiences of social inclusion for people with mental illness were also described at both the family and community levels.

The stigmatising beliefs that Timorese people with mental illness are dangerous, lack capacity and have an incurable illness are consistent with stereotypes that have been reported in both HICs and LMICs [12, 46]. Sometimes these beliefs were related to the sociocultural conceptualisation of mental illness as a sign of intractable ancestral punishment, as has been reported in other parts of Asia [30]. Mental illness may appear intractable to people in Timor-Leste due to the lack of effective mental health care, which means that people may not witness the possibility of recovery [32, 44]. Attributing the cause of mental illness to the family (past and present) and the consequent shame they experience has been described globally [10, 47], and underscores the importance of adopting a family-centred approach to mental health care, stigma reduction and social inclusion in Timor-Leste.

Some features of mental illness can make it difficult for unwell people to fulfil prescribed family and social roles (e.g. lack of concentration and motivation may hinder reciprocal relationships with community members). Because of these unfulfilled social roles, community members may perceive people with mental illness negatively and keep social distance [28]. Timorese people, including extended family members, may also distance themselves from people with mental illness because they fear some illness behaviours (e.g. aggression) [48, 49]. Social distancing towards people with mental illness is seen elsewhere in Asia, with a large study of 960 adults in India finding that participants reported more social distancing when they believed that people with mental illness were dangerous [49]. Specific to Timor-Leste, social distancing may also relate to a fear from family and community members that they will be economically burdened because of the need to contribute (i.e. money or livestock) to healing rituals, or even that they may become ‘spiritually contaminated’ themselves if they are involved in spiritual processes to resolve their family’s history of mental illness. In this setting, rights-based approaches to mental health offer an alternative narrative and approach to support people with mental illness.

The marginalisation and vulnerability of people with mental illness in Timor-Leste was also a prominent finding. Despite protection under the Timorese constitution, people with mental illness experienced multiple forms of discrimination and violence that included bullying, physical and sexual violence, and confinement. These findings echo previous human rights reports emerging from Timor-Leste and globally [1, 50, 51]. Confinement and restraint are explained as ways to protect people with mental illness in many countries [1, 28, 52]. However, research from Indonesia and Ghana demonstrates that families also employ chaining when they feel inadequately supported and have to attend to competing demands [52, 53]. Our findings highlight the urgent need for the Timor-Leste government to provide effective mental health treatment as a strategy for promoting recovery and social inclusion as well as reducing reliance on chaining and confinement.

The stigma, discrimination and social exclusion of people with mental illness in Timor-Leste reflect an unsupportive and restrictive environment cutting across multiple levels. Participants reported that many people with mental illness were excluded from formal employment, educational, social protection and legal systems. Timorese people who are unwell and excluded for lengthy periods often lacked the skills and education required for independent living and employment, which, over time, become increasingly difficult for them to acquire [54]. They may also opt out of activities or not disclose their illness because they anticipate stigmatizing attitudes and discrimination [55]. This underscores the importance of interventions to combat individual, community and systemic forms of discrimination in order to accommodate the rights, needs and perspectives of people with mental illness and their families.

The exclusion of people with mental illness from formal systems may be explained in part by Timor-Leste’s broader development challenges [50]. Despite enormous progress since independence, Timor-Leste’s national employment rate is only 53, and 86% of people with disability in Timor-Leste do not receive the disability pension [37, 40]. Inconsistent legal and political practices regarding people with mental illness may reflect a lack of legislative infrastructure [56], including a lack of clarity in the Timorese constitution regarding the rights of people with mental illness in the absence of UNCRPD ratification [57]. Given the low (under) estimated population proportion of Timorese people with mental illness, promoting their social inclusion may not be a government development priority.

Examples of inclusion of people with mental illness in Timor-Leste were also described. Family and community structures promoted unity, acceptance and responsibility for people with mental illness. The closeness of the community, particularly in rural areas, increased community members’ exposure to and familiarity with people with mental illness, which has been found to reduce stigma and social distancing [49, 58]. People with mental illness were also able to derive social capital by participating in activities surrounding family life and livelihoods (i.e. agricultural and domestic tasks) that contributed to intergenerational well-being and hence actively promoted their sense of belonging [59]. Contribution to livelihoods was identified by people with mental illness in India and Nepal, other communitarian contexts, as an important aspect of social inclusion [26]. Despite their minimal formal involvement in disability persons organisations, some people with mental illness have benefited from participation in the burgeoning disability-rights movement in Timor-Leste, which promotes the availability of the disability pension and CBR course. Inclusion of people with mental illness within the mainstream disability-rights movement is a key focus of development practice throughout the Asia Pacific due to recognition that such movements have tended to concentrate on people with physical or sensory disabilities [60]. In Timor-Leste, existing networks of peers with mental illness could be mobilised to strengthen civil society representation of people with mental illness.

This study has several limitations. There is a risk that participants did not disclose relevant negative experiences, especially to a foreign interviewer, which may mean that we did not capture the full range of experiences. We could not explore the effects of illness type on social exclusion as we did not undertake clinical assessment of participants. Our sample was not representative and was unlikely to encompass the most vulnerable people with mental illness because we recruited people in contact with health and social services, most of whom were from Venilale and Baucau. However, we believe the emphasis on rural-dwelling Timorese was important because most of the population lives outside urban centres. In addition, other stakeholder groups also described the inclusion and exclusion of people with mental illness across Timor-Leste, thereby providing some triangulation of findings.

Future research could employ more participatory methods (e.g. Photovoice) to further engage the voices of Timorese people with mental illness so that they can tell their own stories and use research as a practice of inclusion [61]. Further investigation of the prevalence and extent of these experiences of social exclusion, and to better identify protective factors that promote the social inclusion of people with mental illness in different regions of Timor-Leste is indicated.

## Conclusions

While this article has concentrated on the multi-faceted exclusion of Timorese people with mental illness and their families, there are local cultural strengths that can be harnessed for realising social inclusion. Social inclusion can be promoted by emphasizing the synergies between human rights and Timorese sociocultural values of acceptance, unity and responsibility. There is a clear need to increase population awareness of mental health and illness in general, and strategies for inclusion in particular. Such mental health education should incorporate the sociocultural conceptualisations of mental distress and the priorities of people with mental illness and their families, including practical barriers families face when caring for unwell family members (i.e. time and resource demands). There is an urgent need for the Timor-Leste government to prioritise the development of rights-based mental health services that offer an explanation of, and approach to, mental illness that complements sociocultural practices. Given that health is just one aspect of social inclusion, attention is also needed to ensure that services and systems across sectors are inclusive and accessible for people with mental illness and their families.

## Additional file

Additional file 1: Full Interview Guides. (DOCX 40 kb)

## Data Availability

Participants shared their opinions and experiences upon assurance that their confidentiality and anonymity would be protected. Hence, the research data is not available publicly because this would compromise individual privacy and our ethical approval conditions.

## Abbreviations

CS: Civil society member
DM: Decision maker
FM: Family member
HIC: High-income country
LMIC: Low- and middle-income country
NGO: Non-government organisation
OT: Other community member
PWMI: Person with mental illness
SP: Service provider
UNCRPD: United Nations Convention on the Rights for Persons with Disabilities
WHO IPCHS: WHO Framework on Integrated People-Centred Health Services
WHO: World Health Organisation
